# Crohn’s disease: prevalence, MR features, and clinical significance of enteric and colonic sinus tracts

**DOI:** 10.1007/s00330-020-06935-1

**Published:** 2020-05-26

**Authors:** Martina Scharitzer, Bernd Koizar, Harald Vogelsang, Michael Bergmann, Christian Primas, Michael Weber, Wolfgang Schima, Thomas Mang

**Affiliations:** 1 Department of Biomedical Imaging and Image-guided Therapy, Medical University of Vienna, Waehringer Guertel 18-20, 1090 Vienna, Austria; 2 Department of Internal Medicine I, Klinikum Wels-Grieskirchen, Grieskirchner Straße 42, 4600 Wels, Austria; 3 Department of Internal Medicine III, Division of Gastroenterology & Hepatology, Medical University of Vienna, Waehringer Guertel 18-20, 1090 Vienna, Austria; 4 Department of Surgery, Division of General Surgery, Medical University of Vienna, Waehringer Guertel 18-20, 1090 Vienna, Austria; 5 Department of Diagnostic and Interventional Radiology, KH Goettlicher Heiland, KH der Barmherzigen Schwestern, St. Josef-KH, Dornbacher Straße 20-30, 1170 Vienna, Austria

**Keywords:** Crohn disease, Intestinal fistula, Inflammatory bowel disease, Magnetic resonance imaging, Prevalence

## Abstract

**Objectives:**

Enteric and colonic sinus tracts are inflammatory complications that precede intestinal fistulas in patients with Crohn’s disease (CD). The aim of this study was to retrospectively determine the prevalence, morphologic features, and outcome of sinus tracts using MR imaging.

**Methods:**

A consecutive cohort of 642 patients with known CD, referred for MR enterography or MR enteroclysis (study period 01/2014–09/2019), was evaluated retrospectively for the presence of sinus tracts, their locations, presence and length of coexisting strictures, bowel wall thickness, CDMI score, upstream dilation, and bowel distension. Clinical outcome was assessed using medical records. For metric data, means and standard deviation, as well as one-way ANOVA and Pearson’s correlation coefficient, were calculated.

**Results:**

In 36/642 patients with CD undergoing MRE, 49 sinus tracts (forty in small intestine, nine in left-sided colon) were detected with a prevalence of 6.9% in patients with MR-visible signs of CD (*n* = 519, overall prevalence of 5.6%). Mean segmental bowel wall thickness was 8.9 mm, and mean CDMI score was 9.3. All sinus tracts were located within a stenotic segment, showing mesenteric orientation within the small bowel and upstream dilation in 13 patients. Of 36 patients, 19 underwent immediate surgery and seven developed clinical progression within the segment containing the sinus tract.

**Conclusions:**

Sinus tracts occur in 6.9% of patients with visible signs of CD. They are located within stenotic, severely thickened bowel segments with high MR inflammation scores. Their detection is clinically important, because they indicate a more aggressive phenotype and, if left untreated, may show severe progression.

**Key Points:**

*• Sinus tracts occur in 6.9% of patients with MR-visible signs of Crohn’s disease.*

*• Sinus tracts are a radiological indicator of early penetrating Crohn’s disease, with a high risk of progression, and require dedicated treatment.*

*• Sinus tracts can be recognized by characteristic findings and typically occur in stenotic, severely thickened bowel segments with high MR inflammation scores.*

## Introduction

Sinus tracts represent an inflammatory complication of penetrating Crohn’s disease. Different from fistulas, they are defined by their extension into the mesentery or terminate at fascial planes without communication to another epithelialized surface [1–3]. They are also known as incomplete or initial fistulas [4]. Inflammatory masses or, if infected, abscesses can arise from a sinus tract and are common complications in patients with Crohn’s disease. Although the pathogenesis of fistula formation in this patient group is scarcely understood, the transition of epithelial to mesenchymal cells as a reprogramming process could promote migration and invasion into adjacent tissue [5].

Even if technically accessible, endoscopy has methodical limitations in distinguishing a deep ulcer from a sinus tract or even a fistula, since the narrow distal part of the tract cannot be visualized. In contrast, MRI not only provides important information about the extent and activity of chronic inflammatory bowel disease (IBD) [6] but also allows further assessment of extramural complications. It is, therefore, the preferable cross-sectional imaging modality with which to detect penetrating disease [7].

While most of the available literature on penetrating Crohn’s disease focuses on the perianal phenotype, only a few trials have specifically addressed non-perianal intraabdominal fistulas, with varying reported sensitivities and specificities for the diagnoses by MR-Eg and MR-Ec [8]. To our knowledge, no report exists on intraabdominal sinus tracts, their prevalence, imaging characteristics, and the clinical outcome. However, the identification of patients who have a more aggressive phenotype is essential for the initiation of an adequate therapy and for the need to adhere to a closer follow-up. A failure of detection may lead to inappropriate treatment and can also result in increased morbidity and mortality [9, 10]. The aim of this study was to retrospectively determine the prevalence of sinus tracts on MR-Eg and MR-Ec and to assess the imaging features and the clinical outcome.

## Methods

### Patient population

The study was approved by the Institutional Review Board at the Medical University of Vienna. The need for written, informed consent was waived because of the retrospective design of this study. Based on previous evaluations, we assumed a prevalence of about 4%, with 850 cases required to maintain the 95% confidence interval within the limits of ± 1.3%.

**Fig. 1:**
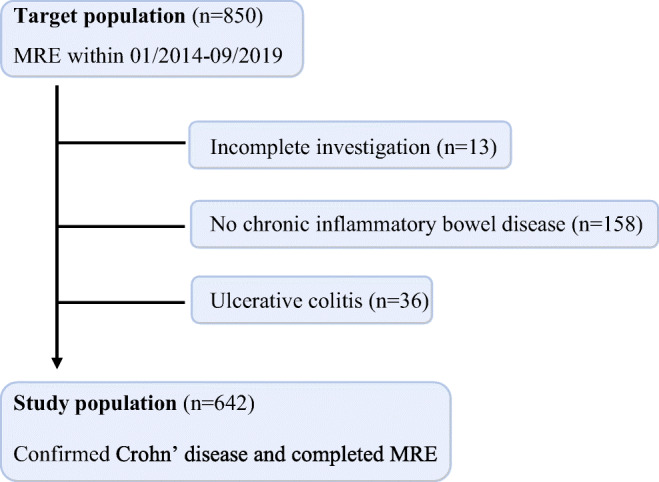
Flowchart for patient inclusion in the study. MRE, magnetic resonance enterography/enteroclysis

In this retrospective longitudinal study, we reviewed all consecutive MR enterography and MR enteroclysis studies performed at our institution between January 2014 and September 2019. Inclusion criteria were (a) MR enterography or MR enteroclysis with intravenous application of contrast medium, performed at our institution for the assessment of inflammatory bowel disease and (b) previous diagnosis or visible signs of Crohn’s disease at MR imaging. Exclusion criteria were (a) non-diagnostic MR studies; (b) MR examinations without IV contrast; and (c) diagnoses other than Crohn’s disease. The final study group included a total of 642 patients. A patient selection flowchart is shown in Fig. 1.

### MRE protocol

MR imaging was acquired with a 1.5-T MR scanner (Magnetom Aera®, Siemens Healthineers) until June 2016 and a 3.0-T MR scanner (Trio Tim, Siemens Healthineers), with an upgrade in June 2017 (Magnetom Prisma fit®, Siemens Healthineers). All patients followed a low-fiber diet and fasted for at least 6 h before imaging. For MR enterography, patients started to drink an oral contrast material solution 45 to 60 min before imaging, which consisted of either water mixed with a powdered soluble dietary fiber (Optifibre®, Nestlé), or a 2.5% mannitol solution to prevent intestinal resorption.

MR examinations were performed in adherence to the European guidelines [11] and included a coronal and axial T2-weighted fast imaging with steady-state free precession, axial and coronal T2-weighted half-Fourier single-shot turbo spin-echo sequences (HASTE) with and without fat saturation, and pre-contrast fat-saturated coronal T1 volumetric interpolated spoiled gradient-echo sequences (VIBE). To reduce bowel peristalsis, 20-mg hyoscine butylbromide (Buscopan®, Boehringer Ingelheim) was administered intravenously in all patients, unless contraindicated. Dynamic, contrast-enhanced, coronal 3D T1-weighted fast-saturated VIBE sequences were acquired 45 and 70 s after the intravenous administration of gadolinium contrast medium (gadobutrol [Gadovist®] 1.0 mmol/L, Bayer Schering Pharma, or gadoterate meglumine [Dotarem®] 0.5 mmol/L, Guerbet), followed by a 10-mL saline flush. Additional axial post-contrast 3D T1-weighted fat-saturated VIBE images were acquired.

### Image analysis

All images were interpreted by two board-certified gastrointestinal radiologists (M.S., T.M.), with more than 10 years of clinical experience in IBD. The two radiologists were blinded to the results of endoscopic and histopathological examinations and clinical follow-up. Consensus among both readers, with repeat review of images, was used to achieve the best possible standard of reference. For the assessment of sinus tracts, the presence of a blind-ended tubular structure that extended into the mesentery without contact with another luminal structure was recorded [1].

Additional MRE features were evaluated according to the CDMI score [12] and included the following: maximum bowel wall thickness next to the sinus tract in millimeter; degree of contrast enhancement (four-point scale); assessment of mural edema on T2-weighted fat-saturated sequences (four-point scale); and assessment of perimural T2 signal (four-point scale).

The presence and the length of a stricture were assessed and reported in centimeter. The following criteria were used to determine whether a stricture was present at the site of a sinus tract: (a) > 80% luminal narrowing compared with adjacent unaffected bowel loops; (b) bowel wall thickness > 3 mm; and (c) persistent narrowing, assessable on all acquired MRE sequences [13]. To assess the upstream dilation, the transverse luminal diameter proximal to the stenotic segment was measured according to recent recommendations (small bowel < 3 cm, 3–4 cm, > 4 cm; large bowel > 6 cm) [3, 14]. To evaluate bowel distension, we used a score published by Bekendam et al including (1) for no distension or collapsed segment (< 25% of segment adequately distended), (2) insufficient distension (25–50%), (3) for sub-optimal distension (50–75%), and (4) for optimal distension (> 75%) [15].

### Statistical analysis

Statistical analysis was performed using IBM SPSS Statistics for Windows Version 26.0 (IBM Corp). Metric data are described as means ± standard deviation if normally distributed, or median (min, max) if skewed. Categorical data are presented using absolute frequencies and percentages. Crosstabs and chi^2^ or Fisher exact tests were used to compare groups with regard to categorical data. One-way ANOVA (given homogenous variances) or Welch corrected the three clinical outcomes for metric data. Students’ unpaired *t* test was used to compare the two different locations regarding metric data. Pearson’s correlation coefficient was calculated to assess the correlation between metric data. Correlation for ordinal data between upstream dilation and the degree of bowel distension was assessed using Spearman’s rank correlation. A *p* value ≤ 0.05 was considered to indicate statistically significant results.

## Results

Among 642 patients with Crohn’s disease and MR studies, 540 patients were investigated with MR enterography and 102 patients with MR enteroclysis. Within this study population, 49 sinus tracts were identified in 36 patients, for an overall prevalence of 5.6% (Table 1). Focusing only on patients with signs of inflammation visible on MRE (*n* = 519), the prevalence of sinus tracts was 6.9%. Nine patients had two sinus tracts, and two patients had three sinus tracts. Imaging characteristics of sinus tracts are shown in Table 2.

**Table 1:** Demographics of patients with sinus tracts

Patient characteristics (*n* = 36)	*n*	%
Sex		
Male	15	41.7
Female	21	58.3
Age at diagnosis		
< 17 years (A1)	2	5.6
17–40 years (A2)	22	61.1
> 40 years (A3)	12	33.3
Prior bowel resection (one or more)		
Yes	8	22.2
No	28	77.8
Concomitant drug(s) at time of MRE		
5-ASA	5	13.8
Thiopurine/methotrexate	10	27.8
Corticosteroids	2	5.6
Anti-TNF	13	36.1
No IBD drugs at baseline	9	25

**Table 2:** Imaging characteristics of sinus tracts (*n* = 36 patients)

Location of sinus tract		
Ileum	5	13.9
Terminal ileum (within 10 cm to ileocecal valve)	24	66.7
Colon	7	19.4
Number of sinus tracts		
1 sinus tract	25	69.4
> 1 sinus tract	11	30.6
Associated fistula	10	27.8
Upstream luminal diameter		
Small bowel (*n* = 29)		
> 4 cm (moderate-severe)	3	
3–4 cm (mild)	9	
< 3 cm (probable)	17	
Colon (*n* = 7)		
< 6 cm	1	
< 6 cm	6	

**Fig. 2:**
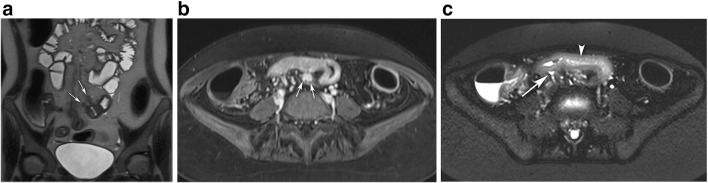
MRI of a 21-year-old man with Crohn’s disease, abdominal pain, and diarrhea. **a** Coronal T2-weighted image shows two sinus tracts of the ileum (arrows) with a characteristic mesenteric orientation within a stenotic bowel segment. On post-contrast T1-weighted fat-saturated imaging (**b**), marked contrast enhancement can be seen (arrows). Axial T2-weighted fat-saturated image (**c**) shows mural edema (arrowhead) and perienteral fluid rim (arrow), resulting in a CDMI score of 10. The patient underwent subsequent surgery with resection of the ileal segment

**Fig. 3:**
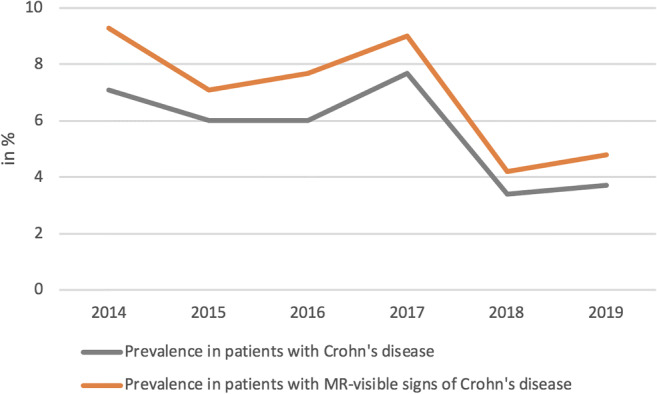
Prevalence of sinus tracts (%) across different years of the study period, stratified by the presence of Crohn’s disease in all patients and in patients with visible signs of Crohn’s disease on MR imaging

The mean CDMI score was 9.25, with slightly higher scores at younger ages (mean CDMI score < 17, 9.00; 17–40, 9.59; > 40, 8.67); although the difference was statistically insignificant (*p* value = 0.152). CDMI scores did not differ significantly between small (CDMI = 9.17) and large bowel (CDMI = 9.57) (*p* value = 0.487). All sinus tracts were located within a constantly narrowed bowel segment with a median length of 9.5 cm (range, 3–28 cm). The length of the stenosis showed a significant but moderate correlation with the CDMI score (*r* = 0.397; *p* value = 0.016). When located within the small bowel (*n* = 29), all sinus tracts showed a mesenteric orientation (Fig. 2). Upstream dilation was found in 13 patients (Table 2), two of them having an MR-Ec. In the remaining 26 patients with a probable stricture, twelve patients had a transverse upstream small bowel diameter of 2.5–3 cm. Two of them had an additional proximal stricture. Upstream dilation showed a significant correlation to the degree of bowel distension of the small bowel (*r* = 0.904; *p* value < 0.001). The prevalence of sinus tracts per year decreased slightly during the study period, although differences were statistically insignificant (Fig. 3, *p* value = 0.665).

### Clinical outcome

Nineteen (52.8%) patients underwent immediate surgery within a median time of 2 months (range, 1–4 months).

**Fig. 4:**
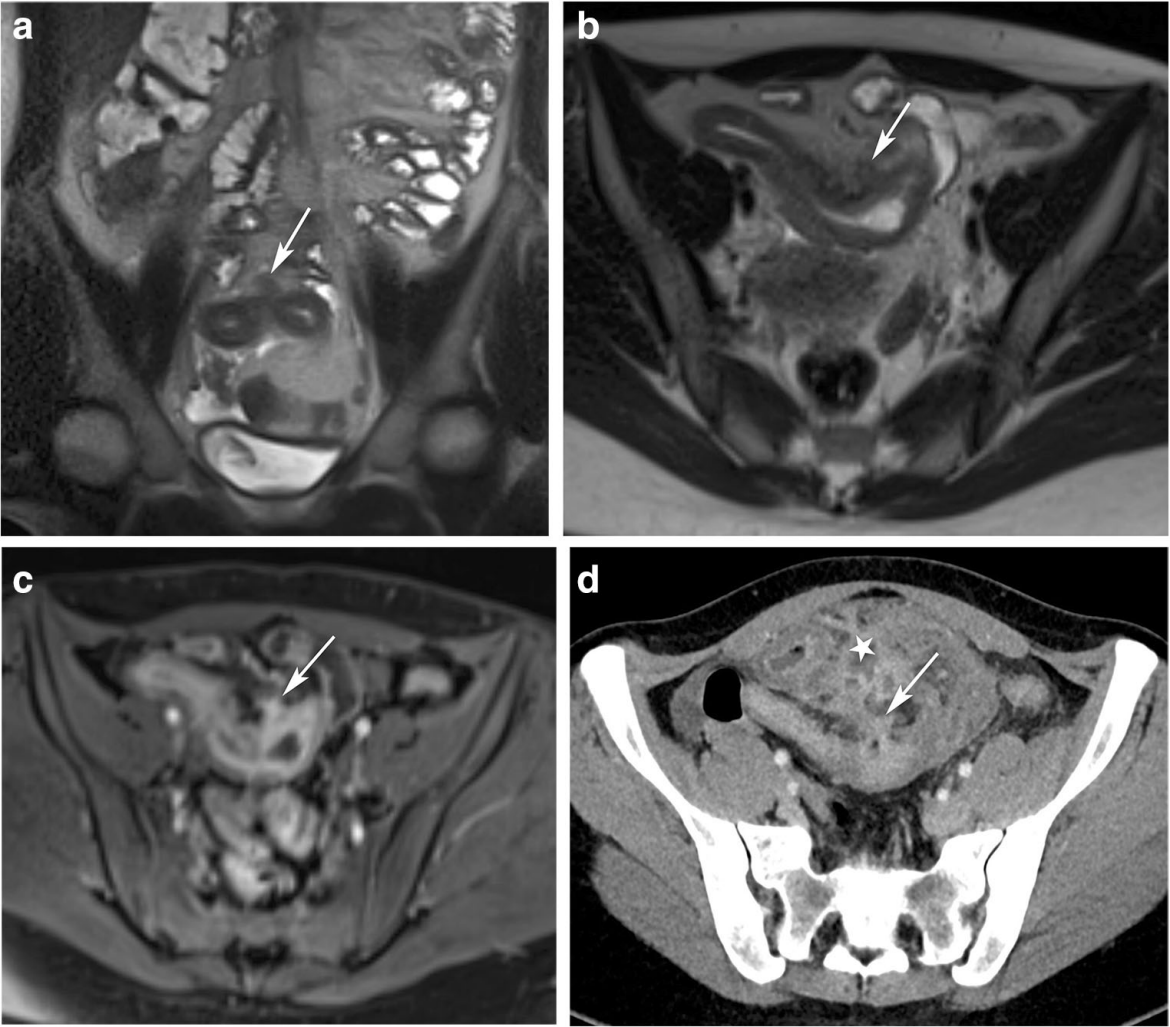
A 26-year-old woman with Crohn’s disease and diarrhea: follow-up of a sinus tract. Coronal (**a**) and axial (**b**) T2-weighted MR images show a single sinus tract within the preterminal ileum (arrow), with marked enhancement of the bowel wall (arrow) on post-contrast imaging (**c**) and a location within a stenotic segment. Eleven months later, the patient presented with an acute abdomen. Axial contrast-enhanced CT image (**d**) shows a large abscess formation (star) originating from the bowel segment that contained the sinus tract (arrow)

Median follow-up for the other patients (*n* = 13) was 19 months (range, 5–37 months). Seven of them (54%) developed progression that originated from the site of the sinus tract within a time interval of 10 months (range, 5–17 months). Complications were either abscess formation (Fig. 4), free perforation, or fistula formation. Further details about the location and number of sinus tracts, type of complication, surgical treatment, and postoperative follow-up of each patient are provided in Table 3. The medication at the time of MR imaging in these patients was Anti-TNF in 3 patients, methotrexate in one patient, corticosteroids and 5-ASA in one patient, and no IBD drugs in two patients. Three patients with conservative treatment received a balloon dilation, since the length of the stenosis at the terminal ileum was less than 4 cm.

**Table 3:** Location and number of sinus tracts, type of surgery performed, and postoperative course of patients with complication originating from the site of the sinus tract (*n* = 7)

Patient ID	Location number of sinus tracts		Type of complication	Operation performed	Postoperative complication
1	Ileum	3	Perforation	Ileocecal resection	Wound dehiscence, reoperation
2	Left colonic flexure	1	Clinical exacerbation with peritonitis	Subtotal colectomy with ileorectal anastomosis	–
3	Neoterminal ileum	1	Abscess formation	Right hemicolectomy with ileotransversostomy	Dehiscence of anastomosis, creation of a jejunostomy
4	Ileum	1	Abscess formation	Ileocecal resection	–
5	Terminal ileum	1	Perforation, enteroenteric fistula formation	Ileocecal resection (70 cm ileum resected, conversion to laparotomy)	–
6	Terminal ileum	1	Enteroappendiceal fistula formation	No operation, close monitoring	
7	Descending colon	1	Enterocutaneous fistula and abscess formation	Segmental resection, new stoma system	–

MRE characteristics associated with clinical outcome are shown in Table 4. The average CDMI scores differed for the three clinical outcomes (immediate surgery, 9.42 ± 1.34; clinical progression, 9.86 ± 0.900; conservative, 7.83 ± 0.983; *p* = 0.012). In addition, there was a statistically significant difference between the three clinical outcomes with regard to the length of the stenoses (immediate surgery, 11.38 ± 6.35 cm; progression, 9.86 ± 4.13 cm; conservative treatment including endoscopic dilation, 4.9 ± 1.70 cm; *p* = 0.002).

**Table 4:** Follow-up of 32 patients with sinus tracts: MRI findings*

	Total	Immediate surgery	Clinical progression	Conservative treatment	*p* value
Wall thickness					
> 7 mm	29	18	7	4	0.076
≤ 7 mm	3	1	0	2	
Location					
Small bowel	26	15	5	6	0.388
Large bowel	6	4	2	0	
Stricture length					
Length ≤ 5 cm	5	1	1	3	0.031
Length > 5 cm	27	18	6	3	
Pre-stenotic dilation					
Not visible	19	10	6	3	0.274
Visible	13	9	1	3	
Fistula					
Coexisting fistula	8	6	2	0	0.288
No coexisting fistula	24	13	5	6	
Number of sinus tracts					
1	23	14	6	3	0.347
> 1	9	5	1	3	
CDMI score (mean)	9.22	9.42	9.86	7.83	0.012

*4/36 patients were lost to follow-up and were excluded

## Discussion

Sinus tracts are an early sign of penetrating Crohn’s disease. They may develop at any anatomic location along the gastrointestinal tract. Sinus tracts are clinically relevant, because they may progress to abscess and fistula formation. While they are well-described in the perianal region, only a limited number of studies are available that address non-perianal penetrating disease. Referral-based studies including patients from tertiary-care centers have shown a higher lifetime risk for the development of internal fistulas, ranging from 20 to 40% [16], whereas population-based studies found that 16% of adults [17] and 2.2% of children [18] developed internal fistulas. Stratification between early penetrating disease and fistula formation has not been reported as yet.

The results of our study have shown sinus tracts in 6.9% of patients with active inflammatory signs of Crohn’s disease at MR imaging. Bruining et al evaluated the prevalence of penetrating disease by analyzing the records of consecutive patients who underwent CT enterography during a time period of 2 years. They reported sinus tracts in 2% and a prevalence of fistula in 20.7% [19]. However, a differentiation between a perianal and a non-perianal location was not reported in this study. The higher prevalence of sinus tracts in our study may be explained by advanced imaging based on MR-Eg and MR-Ec and by the focused, image-based search of radiologists with dedicated expertise in imaging of chronic inflammatory bowel disease. Furthermore, the general awareness of radiologists for sinus tracts may have previously been lower and sinus tracts may have not been targeted, or always recognized as specific findings.

Interestingly, we observed no increase, but even a slight, albeit non-significant decrease in the incidence of sinus tracts over the years of the study period. This is in contrast to a reported increase in the incidence of inflammatory bowel disease with time [20]. It may reflect the fact that rapidly changing improvements in medical therapy have changed the natural history of IBD. This is in line with a meta-analysis that showed a significant reduction in surgery over the past decades, which was attributed to earlier disease detection, increased use of immunomodulators, and anti-TNF therapies [21].

With regard to gender distribution, sinus tracts were more common in female patients (58%), which is consistent with previous reports [22]. The age distribution of our patients is also comparable with that of previous studies. According to Charpentier et al, 78% of patients affected by Crohn’s disease are under 40 years of age [23], while 67% of our patients with a sinus tract were among this age group.

We chose the CDMI score for this evaluation because it has been validated by histopathology [24], does not incorporate the evaluation of ulcers (which would be positive in all our cases), and shows similar interobserver agreement and correlation compared with the MaRIA score [12]. Our patient group with sinus tracts greatly exceeded the published cutoff value of 4.1 for active disease [25], with a median CDMI score of 9.3.

To the best of our knowledge, morphologic aspects of sinus tracts at imaging have not been described previously. In the course of this study, we could identify imaging features typically associated with enteric and colonic sinus tracts with regard to their morphological appearance. All sinus tracts were located within a stenotic bowel segment, with almost half of the patients demonstrating a prestenotic bowel dilatation. Endoluminal pressure and upstream dilation were shown to be contributing factors for penetrating disease [26]. The observed strong association between a stenotic segment and a penetrating course of the disease is supported by both clinical experience and published pathology literature [4, 27] and was also found on cross-sectional imaging in pediatric patients [28]. Previous reports have shown a lower association between penetrating Crohn’s disease and stenoses, with reported simultaneous occurrence in 25% and 47.1% of patients, respectively [29, 30]. This lower association may be related to the fact that the evaluation criteria were not exclusively based on radiological evidence.

In the present study population, patients undergoing surgery as primary therapy after MR imaging had involvement of the terminal ileum in 75%. This is consistent with previous observations by Cerqueira et al, who also reported that risk factors for surgery comprised disease at young age, terminal ileal involvement, and stenotic or stenotic-penetrating behavior [31]. The majority of Crohn surgeries are performed due to stenoses, followed by complications of penetrating Crohn’s disease and others, such as therapy failure, gastrointestinal bleeding, a toxic megacolon, or neoplasia [32].

In addition to clinically significant stenosis, inflammatory masses and abscesses are severe complications of Crohn’s disease. They are found in up to 10–28% of patients with Crohn’s disease, and they can be found in 3.4–4.2% of patients who undergo cross-sectional imaging [33, 34]. We observed a progression to perforation, abscess, or fistula formation, originating from the site of the sinus tract, in seven of 13 patients (54%) without immediate surgical therapy and available follow-up. Sinus tracts, therefore, may act as dedicated indicators of lesions with a more aggressive behavior phenotype and may be interpreted as a warning sign of severe clinical progression. Their presence in a patient with Crohn’s disease may demand modification of the therapy. In patients with perianal fistulas, biologic and immunomodulator therapies are associated with reduced hospitalization and surgical intervention [35]. However, these results may not be generalized to internal fistulas [36, 37]. Therefore, the European Crohn’s and Colitis Organization (ECCO) and the European Society of Colo Proctology (ESCP) stated that, in the case of internal fistulas, due to poorer response to medical therapies, the possibility of surgical removal should always be considered [38]. The high complication rate of sinus tracts in our study would also favor early surgical treatment or the necessity to adhere to close follow-up imaging when choosing biological treatment.

Our study has some limitations. Although we investigated a large patient population over a long study period, due to the rarity of occurrence, we identified only a small number of patients with sinus tracts for the assessment of imaging features and clinical outcome, potentially affecting statistical power. Nevertheless, the image-based evaluation by two specialists may allow extrapolation from the prevalence of our data to a larger population. Since this is a single-center study, the prevalence of sinus tract formation in Crohn’s disease may vary geographically. To the best of our knowledge, however, this is the largest study that specifically addressed enteral sinus tracts and their imaging characteristics and a clinical follow-up of conservative treatment.

Furthermore, four patients were lost to follow-up. This may be attributed to the fact that investigations were performed in a tertiary referral center, which included patients from abroad. Since the loss was < 20% and patients lost to follow-up would not have had a different prognosis, we do not consider this a serious threat to validity.

Finally, the assertion of Solberg et al that more patients with severe disease are treated in specialized centers, where they are more likely to be included in retrospective studies, cannot be refuted [39]. However, because of the increasingly complex management of IBD, particularly for severe cases, admission to high-IBD-volume hospitals has been shown to be associated with better outcomes in both CD and UC [40].

## Conclusion

In 6.9% of patients with MR-visible signs of Crohn’s disease, sinus tracts of the intestine can be found, which are indicative of the penetrating disease type. Characteristic findings include a location in stenotic segments and prestenotic dilation in 13 patients, if located in the small intestine. Correct detection and treatment of sinus tracts are highly relevant to prevent progression to abscess formation. Therefore, radiologic knowledge of this entity and diagnosis at imaging, together with close clinical surveillance or surgical therapy, pose an important interdisciplinary task.

## Abbreviations

5-ASA: 5-Aminosalicylic acid
Anti-TNF: Anti-tumor necrosis factor
CD: Crohn’s disease
CDMI: Crohn’s Disease MRI Index
CT: Computed tomography
ECCO: European Crohn’s and Colitis Organization
ESCP: European Society of Colo-Proctology
HASTE: Half-Fourier single-shot turbo spin-echo
IBD: Inflammatory bowel disease
MARIA: Magnetic Resonance Index of Activity
MR: Magnetic resonance
MR-Ec: MR enteroclysis
MR-Eg: MR enterography
VIBE: Volumetric interpolated spoiled gradient-echo
